# Factors Impeding Health-Care Professionals to Effectively Treat Coronavirus Disease 2019 Patients in Pakistan: A Qualitative Investigation

**DOI:** 10.3389/fpsyg.2020.572450

**Published:** 2020-11-09

**Authors:** Ali Raza, Sheema Matloob, Noor Fareen Abdul Rahim, Hasliza Abdul Halim, Amira Khattak, Noor Hazlina Ahmed, Durr-e- Nayab, Abdul Hakeem, Muhammad Zubair

**Affiliations:** ^1^Business Administration Department, Sukkur IBA University, Sukkur, Pakistan; ^2^School of Management, Universiti Sains Malaysia, Penang, Malaysia; ^3^Graduate School of Business, Universiti Sains Malaysia, Penang, Malaysia; ^4^Prince Sultan University, Riyadh, Saudi Arabia; ^5^Department of English, The Women University Multan, Multan, Pakistan; ^6^Department of Business, National College of Business Administration & Economics, Lahore, Pakistan; ^7^Ghulam Muhammad Mahar Medical College Teaching Hospital, Sukkur, Pakistan

**Keywords:** COVID-19, health-care professionals, Pakistan, obstruction, qualitative investigation

## Abstract

The coronavirus disease 2019 (COVID-19) pandemic, first reported in late December 2019, is regarded as the most significant public health emergency of the century. According to the World Health Organization (WHO), the current outbreak of COVID-19 has affected millions of people and killed hundreds of thousands in more than 200 countries, including Pakistan. Health-care professionals (HCPs) cannot minimize human interactions or isolate themselves from patients due to their jobs and moral duties. Hence, the outbreak needed HCPs to work in adverse and challenging conditions with possible mental health problems. In light of the stated background, this study aims to explore and understand the factors that impede HCPs to effectively treat COVID-19 patients in Karachi, Pakistan. Based on qualitative methods, a phenomenological approach was considered to record the true experiences of HCPs. Twelve doctors and nurses were recruited from five COVID-19 designated hospitals in Karachi, Sindh Province, using purposive and snowball sampling. Semi-structured in-depth telephone interviews were conducted from April 6 to 14, 2020, and analyzed through thematic analysis. The findings suggest that there were two types of constraints, institutional and personal, which were impeding HCPs to treat COVID-19 patients effectively. Institutional constraints include the poor condition of isolation wards, inadequate availability of personal protective equipment (PPE), excessive and uneven workload, and absence of emotional and psychological support in hospitals. Besides, personal constraints include nervousness due to the novel virus, a constant fear of becoming infected, fear of taking virus to family, extreme isolation and loneliness, and feeling of powerlessness. The study found that HCPs in Pakistan have been dealing with a high risk of infection, causing mental health problems such as stress, anxiety, and depressive symptoms. These mental health problems not only affect attention, understanding, and decision-making capacity of HCPs, which could hinder the fight against COVID-19, but they could also have a continuous effect on their overall well-being on a long-term basis. Therefore, the present study outlines important clinical and policy strategies that are needed to support HCPs as the pandemic continues.

## Introduction

The coronavirus, first reported in late December 2019, spread wide in January 2020 as China was preparing to celebrate New Year (World Health Organization [WHO], 2020a). Chinese authorities specified Wuhan City as the source of the virus, specifically the seafood marketplace. Originally named “severe acute respiratory syndrome coronavirus 2” (SARS-CoV-2) due to its genetic similarities to SARS, the World Health Organization (WHO) named it coronavirus disease 2019 (“COVID-19”) on February 11, 2020 (World Health Organization [WHO], 2020b). By the end of January, COVID-19 was announced as a public health emergency; however, on March 11, 2020, it was declared as a worldwide pandemic (Cucinotta and Vanelli, 2020). The current estimates (June 13, 2020) indicate that 7,553,182 people are infected with COVID-19 across the globe. Among them, 423,349 people have lost their lives in the battle against the pandemic (World Health Organization [WHO], 2020c). The unpredictable outbreak and unknown nature of the clinical presentation, changing symptoms, and transmission methods of COVID-19 have caused high psychological fear among common people as well as health-care professionals (HCPs) fighting as frontline workers (Berger et al., 2020; Rana et al., 2020; Xiao et al., 2020).

Pakistan reported the first confirmed COVID-19 case on February 26, 2020, in Karachi. The Ministry of National Health Services Regulations and Coordination (2020) confirmed 139,230 COVID-19 cases with 2,632 deaths on June 14, 2020. Pakistan has raised concerns that the nation may be the next to be hit hard by the pandemic unless effective and timely steps are taken. Moreover, WHO has warned Pakistan for an increase of 200,000 cases by mid of July, as the virus has already spread over 115 districts mainly in two provinces, Punjab and Sindh (The News International, 2020). Thousands of HCPs are at the frontline of the pandemic, but a shortage of personal protective equipment (PPE) and other medical facilities has subjected them to the risk of the disease (Aamir Latif, 2020).

Since WHO has ranked Pakistan as 122nd among 191 countries in overall quality of health-care systems, for inadequate health infrastructure and HCPs (Tandon et al., 2000; Sadiq et al., 2019; World Health Organization [WHO], 2020c), the country is placed 154th out of 191 countries in global Healthcare Access and Quality (HAQ) index where the burden of disease is high (Fullman et al., 2018; Murray et al., 2018). In addition, the Human Development Index (HDI) value is as low as 0.56, which positioned the nation at 152nd out of 189 countries (United Nations Development Program [UNDP], 2019). In this serious situation, when the health system is already weaker in catering to the needs of the country’s 208.8 million population, the advent of COVID-19 is unfortunate. HCPs are always there on the frontline for the elective treatment and urgent medical care for COVID-19. It makes HCPs at the most significant risk for being infected (Ali et al., 2020).

In Pakistan, the sudden surge of COVID-19 infections and deaths of HCPs was worrying. In April, 480 HCPs were infected, and five doctors died since the COVID-19 outbreak hit the country on February 26 (Gul, 2020). The official sources stated that the exact number of HCPs affected was still unknown. The exponential rise in infections raises the safety and prevention concerns among HCPs, and they refused to perform their duties in COVID-19 wards and emergency units. Moreover, the situation became more concentrated when around 150 doctors and nurses called for strike in Quetta city against the poor working conditions and lack of medical supplies, e.g., PPE. In addition, the HCPs were raising their voices about the inability of the government and health department to deal with the pandemic and for carelessly risking the HCPs’ lives at large (Hashim, 2020; Khan, 2020). Instead of listening to their concerns, the state authorities used physical force to disperse strikers. Furthermore, the government allowed the state authorities to use tear gas and to beat up strikers with sticks and fists in which many doctors were injured. In addition, more than two dozen HCPs were arrested (Khan, 2020).

The state authorities’ actions were shocking for the entire nation, as in many countries around, the world people came out to the streets to applaud their health workers during the crises. The adverse behavior on HCPs causes even more mental health problems affecting the efficiency and effectiveness of their work and has long-term harmful effects on their overall well-being (World Health Organization [WHO], 2005; Lima et al., 2020). Banerjee (2020) stated that addressing the mental health issues in medical workers is thus crucial for the better prevention and control of the pandemic. In Pakistan, several online comments are calling for the frightening state of HCPs working in the isolation wards. However, to the best of our knowledge, no systematic qualitative research has been conducted to address the urgent issue. In the view of the scenario as mentioned earlier, the present study aimed to explore and understand the factors that might have caused the HCPs to resist serving in COVID-19 isolation wards/emergency units and effectively treat COVID-19 patients in Pakistan.

## Materials and Methods

### Research Approach

In the present study, the phenomenological approach was used to obtain rich experiences of the doctors and nurses who had firsthand knowledge and experience of the situation. The phenomenological approach allows exploring and understanding in-depth the lived experiences of the phenomenon with a retrospective view (Salmon, 2012).

### Study Design and Settings

Exploratory qualitative research under the phenomenological approach was considered appropriate to address the main research objective(s). The qualitative inquiry provides more in-depth and broader insights into the phenomena that might have remained unnoticed by survey-based research methods (Punch, 2013). The participants of this study include doctors and nurses who were working in the COVID-19 wards/emergency units and had direct contact with confirmed and suspected COVID-19 patients for at least 2 weeks. The study participants were chosen regardless of their experiences and medical specialty. The study was conducted in Karachi—a cosmopolitan city and the largest city with a population of more than 30 million in Sindh Province. The first COVID-19 case was confirmed on February 26, 2020, in Karachi, Sindh Province. Within 15 days, the number of total confirmed cases (COVID-19 positive) reached 20 out of 471 suspected cases, with the highest numbers in Sindh Province in Pakistan (National Institute of Health Sciences [NIHS], 2020). Besides, 25% of the recorded COVID-19 cases and deaths in Pakistan have been reported in Karachi. It has emerged as the most-affected city of Pakistan (Gulf Times, 2020). At the time of the investigation, Karachi city reported 38,515 (May 14, 2020), which was the highest COVID-19 confirmed cases among other cities in Sindh Province (Health Department, 2020). It is essential to mention that Sindh Province was also among the most affected provinces of Pakistan, having 129,179 COVID-19 confirmed cases in comparison with Punjab with 96,036, Khyber Pakhtunkhwa (KPK) 35,293, Baluchistan 12,742, Islamabad (Federal) 15,578, Gilgit-Baltistan 2,816, and Azad Kashmir 2,277 (Health Department, 2020). In addition, most HCPs were infected with COVID-19 virus in Sindh with 1,804 including 1,626 doctors and 178 nurses. Also, at the time of the advent of the COVID-19 outbreak in Pakistan, Karachi was the only city in Sindh Province that designated few hospitals to deal with COVID-19 patients, and no other cities had the facility for treating COVID-19 patients (Government of Sindh, 2020).

On April 2020, there were only seven designated hospitals in Karachi (both public and private) that established temporary isolation wards inside and outside of hospitals to treat the increasing number of COVID-19 cases. The intention was to target those hospitals that had maximum number of HCPs. Out of seven, five hospitals agreed to participate in the study. Among the five, three were government hospitals and two were private hospitals. The remaining two hospitals declined to participate in the study, as their research departments were looking into research matters. The detailed characteristics of selected hospitals are shown in Table 1.

**TABLE 1:** Hospitals characteristics.

**No.**	**Hospitals**	**Number of beds in isolation wards**	**Total number of ICU beds**	Total number of HCPs working in isolation wards (approximate figures)*	**Number of participating HCPs**
**Government-run hospitals authorized for admitting COVID-19 patients (Karachi)**					
1	Hospital A	48	10	Total doctors: 17 (8 hours rotation) Nurses: 10	03
2	Hospital B	50	28	Doctors: 21 (8 hours rotation) Nurses: 10	02
3.	Hospital C	65	12	Total doctors: 25 (8–10 hours rotation) Nurses: 10	04
**Private hospitals authorized for admitting COVID-19 patients**					
1	Hospital D	40	20	Doctors: 20 Nurses: 11–15	1
2	Hospital E	45	30	Doctors: 11 Nurses: 11–15	2

Source: Health Department (2020). *Data directly obtained from respective hospitals.

The participating hospitals only allowed to contact HCPs on the phone rather than face-to-face meetings. In doing so, the hospital management provided phone numbers of willing participants. The researchers initially contacted HCPs through text messages to ask for their convenient time for the interview. The researchers reassured all participants that their involvement is voluntary and that they have the right to withdraw from the study at any point without stating any justification. They were also assured that their responses would be kept confidential and that the results of the study will be reported in a collective report form. For the present study, ethical approval was received by the Ethical Review Board of NCBA&E under reference number NCBAE-RYK/REF/20/474.

### Data Collection and Procedures

Semi-structured in-depth telephone interviews were conducted with doctors and nurses. The interview guide was developed based on the review of recent preliminary studies (see Mukhatiar, 2020; Rana et al., 2020). The review of the literature shows a very limited original research related to challenges faced by HCPs during the COVID-19 outbreak (in April 2020), and the majority of these studies were at preliminary stages. Specifically, in the context of Pakistan, there was no single original research study found during searching of the literature review in search engines using multiple keywords, which evidences severe dearth of original research. Furthermore, the novel situation instigated the researchers to conduct the original study to address the matter, but the notion was still emerging in that context at the time when data were collected for this research. Hence, it was not possible for us to detail the predetermined list of themes; instead, we allowed data-driven themes to emerge that facilitated us to obtain the rich experiences of HCPs from the interviews, which served the core purpose of the qualitative inquiry. Therefore, the interview guide with open-ended questions was prepared. The summary of interview topics or domains is detailed in Table 2.

**TABLE 2:** Summary of the interview topics/domains.

**No.**	**Summary of the interview topic guides**
1	Personal feelings and experiences while working in the isolation wards/ICUs.
2	Problems and challenges faced by HCPs in treating COVID-19 patients.
3	Future directions (What key steps should be urgently taken).

The sample size was determined by theoretical sampling; i.e., at the point where no new themes from participants’ experiences emerged, data collection was stopped. Theoretical sampling was achieved after 12 interviews; however, two additional interviews were conducted to observe if any new themes were emerging (Creswell and Poth, 2016). To access the participants, both purposive and snowball sampling techniques were used to obtain the rich and diverse experiences of the HCPs. Here, it is also important to mention that unlike other qualitative studies, which are conducted in everyday settings, this study was conducted in the emergency times and very chaotic situations. The countrywide lockdown, fear of the highly contagious virus, and difficulty in getting access to hospitals and HCPs made the fieldwork challenging. Despite these conditions, we managed to conduct 12 with two additional interviews.

The participants were initially contacted through SMS/WhatsApp rather than a direct phone call to ensure their privacy. In the preliminary conversation in the text messages, we introduced ourselves and the main reason for the contact, and we requested for the convenient time for the interview. Once the initial contact was developed, we started the phone call with greetings and by thanking them for their valuable time despite their hectic schedules. We also repeated the purpose of the contact, e.g., the main aim of the research study, and assured them that their identities and responses would be kept strictly confidential. Furthermore, we explained that the call would be recorded for analytical purposes.

Nevertheless, the audio file will be deleted immediately once the research process is completed. We initiated with the broad question “Can you please tell me about your experience of working in the isolation ward or taking care of COVID-19 patients?” Further questions were asked, for example, how did you feel on the first day? How are your feelings now? What challenges did you encounter? How did you respond? What is the response of hospitals regarding those challenges? What kind of support did you receive? In this process, we carefully used the probes, e.g., please tell me more and why/how/when, to promote in-depth discussion. In the end, we expressed our appreciation to them for their incredible and matchless contribution during the pandemic situation. Also, we sincerely thanked them for sharing their stories genuinely to us. Once again, we reminded and reassured them that all conversations would be kept confidential and ensured our availability by providing our contact details for further information or questions. Each interview was conducted in Urdu, the national language of Pakistan, and lasted for at least 30–40 min. The authors conducted all interviews between April 6 and 14, 2020.

### Analysis

The interviews were analyzed using the Braun and Clarke (2006) method of thematic analysis. Each interview was transcribed into Urdu and translated into English. Data analysis occurred concurrently with data collection, and the transcriptions of each interview were completed within 24 hours of the interviews. All the transcripts were reviewed twice before the first transcript was imported into Atlas.ti 8.03. To validate the findings, the researchers tried to eliminate the subjectivity biasness by assigning a single task to two researchers. This practice was done for interviews and analysis. The analyses from two different researchers were matched for internal validation (congruity purpose). The remaining co-authors reviewed the generated themes to ensure that they are truly reflective of the content of the interviews. In addition, a mutual consensus was reached among all assigned research team members.

## Results

### Participants

A total of 27 participants consisting of doctors and nurses were approached and screened for set inclusive criteria of the study. Out of 27 participants, five did not meet the inclusion criteria. Of 22 remaining eligible participants, four declined to participate in the study. Finally, 18 participants agreed to participate in the study. Among the 18, there were 10 physicians and 8 nurses. However, the researchers reached the point of saturation on the 12th interview. The mean age of participants was 31.5 years. There were eight male and four female participants. A majority of the participants (9/12) were working in the public sector, while the rest were associated with the private health-care sector. The mean experience of the participants was 2.9 years. The participants joined the COVID-19 isolation wards from early March, around 15–34 days before the interviews were conducted. The demographic distribution of the study participants is detailed in Table 3.

**TABLE 3:** Participants profile.

**Characteristics**	**Frequency**
**Participants**	
Nurses	5
Physicians	7
**Gender**	
Male	8
Female	4
**Age group**	
25–30	6
31–35	6
>40	
**Participants service sector**	
Public	9
Private	3
**Service experience (years)**	
1–5	12
6–10	
>10	
**Working days in isolation wards before interview (days)**	
1–10	
11–20	5
>20	7

## Findings

**FIGURE 1:**
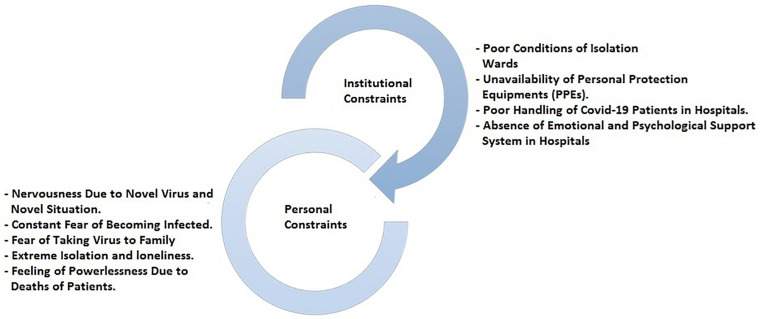
Personal and institutional constraints.

The findings show that all participants were highly committed to take an active part in the battle against the COVID-19 pandemic. The thematic analysis of the interviews resulted in two major themes or categories. The major themes emerged were institutional and personal constraints (especially fear), which were impeding HCPs to perform their jobs effectively (Figure 1). The findings show similar responses from the private and public sectors. The themes generated from the interviews show no significant differences mainly because location, conditions, and constraints associated with dealing COVID-19 patients were the same.

### Institutional Constraints for Health-Care Professionals in Treating Coronavirus Disease 2019 Patients

#### Poor Conditions of Isolation Wards

The majority of the participants was worried about the poor condition of isolation wards and considered it as a significant obstacle in following the standard operating procedures (SOPs) in handling COVID-19 patients:

*I cannot stand there for long. There are no hygiene measures; uneven floor, broken windows, and lack of equipment. How can I treat the patient in such conditions?* (D1)

The findings show that most of the state-run isolation wards were built in unrestrained buildings or non-functional departments of hospitals. In addition, schools, hostels, and labor colonies were used to accommodate COVID-19 patients. According to the participants, these isolation wards lacked the necessary facilities endangering the lives of patients and HCPs:

*There is no common room for us where we could wear protective suits or disinfect us before or after the duty. I used to wear all my protective gears outside the building in open sunlight and then walk to the building in extremely hot weather.* (N2)

Besides, the study found that some isolation centers were set up inside the hospitals by modifying the normal wards for COVID-19 patients. These isolation wards did not meet the criteria or SOPs given by WHO for handling COVID-19 patients. The participants mentioned that the government was not proactive at the beginning of the COVID-19 outbreak in Pakistan. They were very late in disseminating information and official directives, and in allocating the necessary financial budget. Therefore, the sudden surge in COVID-19 patients in the country, especially in Karachi, has created a panic in the health-care sectors and failed to provide any standard facility for the patients. Going through these situations, all participants found themselves in mental distress and extreme pressure for contaminated and unsafe working conditions. On the other hand, the participants working in the private sector were far from satisfied with the facilities, e.g., infrastructure of wards, in that they cannot treat some of the patients:

*We only have 40 beds in isolation wards and few beds in ICU for the COVID-19 patients; which clearly are not sufficient for the velocity of new cases. The patients need to pay a high price ranging from Rs.100,000 to 150,000 per day, and only rich people can avail this facility.* (D4)

The participants disclosed that the private sector provides the patients with premium services, but only a handful can afford and make use of them. In addition, the participants expressed deep concerns on the unrealistic charges per day for COVID-19 patient. They expressed that only elites can avail that service. Also, the participants were not happy with the safety precautions they were presented with.

#### Fighting as Frontline Soldiers Against the Pandemic With No Weapons and Defense Mechanism

The study found shortage or unavailability of PPE as one of the major causes of HCPs’ frustration and distress. The majority of the participants considered this situation “insane,” as many of them were putting their lives at significant risk:

*We have no idea how many patients we have infected or will infect. There are severe stress and fear among us. Then we decided to protest for PPEs not only for ourselves but to save lives of others.* (D5)

The participants said, at early stages, that they used regular glasses, sports goggles, and plastic sheets to protect the face and plastic bags as the gown, risking their lives to treat COVID-19 patients. The participants witnessed many of their colleagues getting infected with COVID-19 infection due to lack of administrative support.

*I lost one of my best friends married just a month ago, due to virus infection because there were no sufficient PPEs available. I wrapped his body in a plastic bag, disinfected him and buried without a proper funeral process. I cannot forget that moment. I am exhausted.* (N2)

Since PPE was unavailable and the working conditions worsened, the majority of health-care employees refused to serve COVID-19 patients and protested against the government and the concerned department.

In response, the participants recalled of the incident that took place in Peshawar, KPK Province, where police assaulted the HCPs who protested for PPE and vulnerable working conditions:

*I cannot imagine how police can do this to HCPs. In the morning, they saluted them for leading the battle against COVID-19, and when they complained about PPEs, police used physical force on them. We cannot find this type of offence in the world.* (D7)

In addition, the HCPs expressed the most profound concern over a debate on social media regarding their refusal to conduct duties and challenge their loyalty with the profession in this critical situation:

*Do I need to go on the suicide mission to prove my loyalty as a doctor? What if, all of the HCPs get infected? Who will be treating the patients? The authorities are hiding their incompetence by blaming the doctors and nurses, which is an offence.* (D5)

Moreover, the participants reported that the PPE that they received was of poor quality. They further briefed that the items (masks, gowns, and shoes) they are receiving are of substandard:

*HCPs infected despite wearing the proper PPEs and following the SOPs. This thing also created a sense of fear among HCPs and doubt about the quality and effectiveness of PPEs.* (N5)

#### Poor Handling of Coronavirus Disease 2019 Patients in Hospitals

Due to lockdown measures in the country, the outpatient departments (OPDs) were closed in all hospitals nationwide. However, emergency departments were open to deal with the normal flow of visiting patients to hospitals. We were informed that the emergency departments have no proper setup or a separate desk for suspected COVID-19 patients at initial screening. Once they were confirmed as COVID-19 patients, they were then transferred to isolation wards. The participants stated that the entire process of dealing with COVID-19 patients in these hospitals is an open threat to the entire crowd of the hospital:

*I see the patients using the same gate of the hospital or even departments for entrance and exit. Moreover, we cannot identify the COVID-19 patients by simply checking their temperature at gates. Some have very mild symptoms. The handling is poor; rather, I say there is criminal negligence.* (D1)

The participants revealed that the majority of the patients are illiterate and belong to low-income families. They are scared to provide correct information about their illness. For example, they hide their symptoms and their travel history and provide misleading information about their previous contacts and families:

*The parents came to our hospital for their daughter’s sickness as she had a consistent cough. They told me that she has a dust allergy and has a history of asthma. While on screening, the patient was found positive for the COVID-19. Later, we came to know that she was living with her husband, who recently came from Dubai and is also sick at home.* (N1)

The study found that patients who are asymptomatic or have mild symptoms are real threats to the HCP*s.*

#### A Never-Ending Fight; Excessive and Uneven Workload

The participants illustrated that they are exhausted due to the overwhelming workload in isolation wards and intensive care units (ICUs). Unlike the regular wards, many uncertainties prolong their stay and duties:

*When I get back from my shift, I am exhausted and cannot figure out how many days or nights have passed on.* (D2)

The participants indicated that some patients become unexpectedly seriously ill and therefore require mental and psychological help too. All these create stress and extra burden for HCPs, as they have been given the responsibility to maintain a positive and healthy environment in the ward:

*Patients with COVID-19 are isolated and have not seen their families for a long time. Thus, we are the main and only point of contact. We often spend our time to make them relax in critical situations regardless of our own mental state.* (N5)

Obligations for staff include not just the additional workload created by such pandemics but also concerns of infection for themselves and their families, dealing with modern and continuously changing measures and PPE, taking care of patients who are severely ill and taking good care of colleagues who have already fallen ill.

#### Absence of Emotional and Psychological Support System in Hospitals

All participants reported that hospitals do not have any interventions or help, which could provide psychological and social assistance to HCPs in COVID-19 pandemic. Moreover, there is no one to listen to them and help out with present fears, anxiety, and stress:

*Literally no one ever thinks of what we are facing in our daily lives. There is no actual channel or helpline for psychologically drained health workers.* (D7)

Another participant expressed:

*In our case, we are struggling to get necessary logistics; getting psychological help in this situation is considered a luxury for many authorities.* (D6)

The study found that HCPs were also struggling to attain sufficient support from family members due to the risk of infection involved. The pressure from family further causes depression and lack of confidence among them. However, the participants reported that they often get psychological support from their colleagues and discuss their problems with them.

### Personal Constraints

#### Nervousness Due to the Novel Virus

The participants revealed that, initially, they were very nervous and lacked the confidence to treat patients with COVID-19. They stated the novel nature of the “virus” itself and its unknown nature, properties, and behavior as some of the major causes of their nervousness:

*The virus is still in the discovery phase, and there is no enough information available regarding its risk, transmission, pathogenicity and treatment.* (D5)

Here, the participants discussed that they are relatively young and had no previous experience of working in ICU or isolation wards under such pandemic situations. In addition, the researchers found that most of the specialist doctors were elderly persons who were more vulnerable to infection. Therefore, the hospital administration did not allow them to have direct contact with COVID-19 patients. However, they were continuously in contact with HCPs in isolation wards.

#### Constant Fear of Becoming Infected

The participants informed that they are hyperactive and ensure that they must not catch the virus infection. This constant mental stress accelerates the state of fear and anxiety while doing duty in the isolation wards.

*Walking into a hall of 100 COVID-19 positive patients brings a lot of uncertainty and fear among* HCPs. *The only certainty in our lives is “Virus” itself because it is definitely in the air, on the furniture we sit, on the equipment we use and even every surface we touch there. We all know the virus does not discriminate.* (N3)

Besides, they reported inadequate health-care facilities in the isolation wards that increase the risk of being infected. Moreover, the study found that the HCPs were concerned as most of the doctors and nurses have been infected with the virus.

The safety of the HCPs should be the top priority because if frontline doctors and nurses were infected, they would become a potential risk for others and patients. Unfortunately, there was a lack of sufficient testing of HCPs who are at high risk of contracting the infection.

They discussed that there is very “little” testing for health-care workers, especially for those who show symptoms:

*We should be tested at least once a week to make sure that we are not vectors for spreading this infection.* (D1)

The participants emphasized on “aggressive testing” of COVID-19 for HCPs as the solution to mitigate their mental distress. If not, these HCPs can be a significant source of virus dissemination across the population.

#### Fear of Taking Virus to Family

HCPs working in the emergency unit reported the feeling of interpersonal isolation and the fear of passing the virus to their families. They expressed serious concerns and fear when they return to home from work:

*I am confident about my fight with multiple fears in the hospital. But when it comes to my family, I am afraid about the consequences.* (N4)

Another participant expressed:

*When I go back home, I throw my clothes in the hamper, run to shower, disinfect all my belongings including, keys, cell phone and even doorknobs, everything. Still, I try not to touch my children which is very painful sometimes.* (D1)

The majority of the participants revealed they are residents of a small apartment, and they do not have any space to self-isolate after the hospital duty. In addition, they could not rent another apartment or a room due to the financial constraints and dependents at their homes:

*In Karachi, the apartment rents are so high, one small unfurnished apartment rents about Rs.10,000. How can I afford it with a low salary and for how much time? Still, we do not know when this pandemic will be over.* (D4)

Treating COVID-19 patients has affected individual HCPs lives, especially females, to purposely take volunteer leave from work due to pressure from their immediate family to avoid any direct contact from virus carrier:

*In our hospital, medical staff includes nurses and doctors were warned by their husbands not to treat the COVID-19 patients otherwise, they will not be allowed to come home.* (N1)

It must be noticed that such social issues have the potential to weaken the health-care system treating COVID-19 patients in the country.

#### Extreme Isolation and Loneliness

The participants expressed their feelings of being isolated all the time. They are supposed to keep a distance from the family, friends, and even their colleagues so that they may not be a source of transmitting the infection to their social circle:

*I feel I am in the incubator. No one really wants to be around me knowing that I am treating COVID-19 patients.* (D5)

#### Feeling of Powerlessness

The participants expressed the feeling of “powerlessness” because they cannot save people from dying:

*As soon as we get to work, you never know what is coming next. In a moment, everything looks so fine, charming, and hopeful. In next, everything is just simply opposite, i.e., respiratory failure, and unfortunate sudden death.* (D7)

Another participant shared:

*It is tough to see or even imagine people dying from virus and their infected bodies wrapped around.* (N3)

## Discussion and Concluding Remarks

The present study highlights HCPs’ lived experiences of battling the COVID-19 pandemic in Pakistan. The findings of the study revealed that the HCPs are working under extreme pressures and making tough decisions. The complex decisions mainly revolve around balancing their physical and mental health-care needs with those of patients and providing care for all unwell patients with inadequate resources resulting mainly in mental health problems (Greenberg et al., 2020).

The participants expressed their deep concerns over unavailability of PPE, deprived conditions of isolation wards, and insufficient supplies. The shortage of PPE, protective gears, and other medical supplies is a global phenomenon and not only in Pakistan, which is a worrying factor in the current scenario. Although Pakistan, being a developing country, is in more critical condition, developed countries are also facing the same issues. There is a lack of adequate PPE, face shields, gowns, and hand sanitizer in the United States, and health-care workers in Italy experienced high rates of infection and death partly because of inadequate access to PPE (Ranney et al., 2020). Similarly, the findings of the study revealed that there is a lack of proper infrastructure to effectively treat COVID-19 patients and the administration flaws in the handling of COVID-19 patients in hospitals. The government has established isolation wards and quarantine areas in teaching hospitals, but these hospitals violate COVID-19 preparedness guidelines (Saqlain et al., 2020a,b). HCPs working in risky conditions experience physical distress and decreased immunity that result in different psychological disorders (Huang et al., 2020).

Considering these issues, it is important for hospital management and relevant authorities to arrange the necessary medical supplies even before letting HCPs into the isolation wards or emergency units. In this context, the previous research shows that during the SARS epidemic, infection control initiatives and higher level of trusts on equipment, e.g., PPE and medical supplies, were related to lower levels of emotional exhaustion (Chong et al., 2004; Marjanovic et al., 2007). In addition, government and hospital authorities must ensure that the isolation wards and emergency units for COVID-19 patients must adhere with the guidelines of WHO. The better working conditions improve the efficiency of HCPs, reduce the state of fear, and lessen the chance of mental distress. The proposed recommendation was also supported by the study of Adams and Walls (2020), who argued that the monitoring and supervision of infection prevention with control measures, reasonable working hour, and appropriate shifts arrangement are key to prevent HCPs from the burnout.

In the current study, psychological factors such as fear of the contracting virus, fear of taking virus to home, and nervousness were also identified as the major themes. These findings are confirmed by very recent studies conducted across the world (see Naushad et al., 2019; Spoorthy et al., 2020; Zhang et al., 2020). As mentioned by Mukhatiar (2020) and Grover et al. (2020), anxiety of falling sick or fear of death could make people hopeless and burnt out. HCPs are facing the worst fear due to their direct contact with the COVID-19 patients by being on the frontline. As found by Zhang et al. (2020), medical health workers had a higher prevalence of psychological problems and risk factors for developing them than non-medical health workers. In this same context, the findings also witnessed that the hospitals mostly lack the facility of psychological support for HCPs. As discussed earlier, the poor conditions at hospitals heightened the risk and fear among the HCPs and infecting their families. Considering these issues, sound infection prevention practices are needed to provide a safe and secure working environment. HCPs who lived at home have concerns about transmitting the virus to family members, which needs to be addressed by hospital administration. One way is to provide separate living accommodation (Adams and Walls, 2020) or financial assistance to secure the family from the unknown virus.

Undoubtedly, the advent of COVID-19 in Pakistan brings various serious challenges for HCPs who are on the frontline. However, these challenges were exponential for the young and junior HCPs who had few clinical experiences in infectious intensive care and belonged to different specialties. The deployment of young HCPs was due to the virus itself, as the older adults are most vulnerable to the worse effect of infection (Vox, 2020). In this study, the participating HCPs’ mean age was 31.5 years and had experience of 2.9 years, which shows that they have noticeably less experience than senior specialists and consultants. The findings suggest the HCPs explicitly expressed the sense of powerlessness about their patients suffering and the loss of lives. They also expressed their fears, lack of management, and problems in emotional stability. These findings are confirmed by the study of Mamas (2020), who stated that junior doctors were moved from being trained to delivery service, and their placement is at greater risk.

Furthermore, Mamas (2020) pointed out that over 100 doctors have died during the COVID-19 pandemic in Italy. In Spain, 20% of those infected with COVID-19 are the individuals who work in the health services. Similarly, in reports in China, the United States, and the United Kingdom, many HCPs have died due to COVID-19 infection. Here, we do not undervalue the novel and contagious nature of the virus and shortages of PPE, but it is a greater risk that junior doctors were deployed in the areas that they may not be familiar with (Mamas, 2020).

Moreover, it is argued that medical equipment such as ventilators is irrelevant when the doctors do not know about their proper usage. Therefore, it is imperative to familiarize with the necessary skill set even there is a lower risk environment and data to practice to perform immediate procedures in the emergency units. In a similar context, the most recent study, i.e., a case report (see Ramachandran, 2020), shared the experiences of one of the junior doctors who shared the story about treating patients to becoming a patient of COVID-19. The reports state that the junior doctor, even though he was at the start of a medical career, showed lack of control and difficulties in information processing. It may be caused by fatigue. This situation strongly indicates the junior or young doctors were not fully ready to handle the outbreak of infectious diseases and required substantial training, education, and improved communication (Huang et al., 2020).

Undoubtedly, at the start of a medical career, high mortality was seen in patients; sudden deaths and no standard treatment were the most significant challenges that shake their confidence. In addition, with the communication challenges posed by strict limitations on family visits, junior doctors should receive additional training and support in breaking bad news (Coughlan et al., 2020). Well-being is particularly crucial for deployed junior HCPs, and simple measures such as introducing junior doctor forums can provide trainees with a space to reflect on stressful experiences with their peers. Despite the considerable disruption to postgraduate training and education, deployment to critical care offers unique opportunities for clinical and professional development (World Health Organization [WHO], 2013). Senior support can help junior doctors acquire transferable skills that will enhance their performance in any field of medicine (Charles and Kumar, 2020).

In the end, it is essential to note that when HCPs become sick, it incapacitates their whole ability and effort to curb the outbreak in the country. During SARS and Middle East respiratory syndrome (MERS) epidemics, HCPs were at higher risk of mental health problems and suffering from post-traumatic stress disorder after the epidemic (see Maunder et al., 2003; Marjanovic et al., 2007; Lee et al., 2018). There is a need to properly prepare staff for the associated challenges to reduce the risk of mental health problems through various mechanisms. As suggested by Greenberg et al. (2020), routine support processes (such as peer support programs) should be made available to the medical staff workers. Furthermore, HCPs require health protection and adequate working conditions, e.g., provision of necessary and sufficient medical protective equipment, the arrangement of adequate rest, and “recovery programs aimed at empowering resilience and psychological well-being” (Zhang et al., 2020, p. 8). Adams and Walls (2020) suggested a supportive system for the health-care workers, for example, ensuring that workers feel they get adequate rest, provision of food, and rest breaks. Results of the recent study suggested that the social support given to medical staff caused a reduction in anxiety and stress levels (Xiao et al., 2020). Urgently, hospitals and relevant authorities need to monitor HCPs mental health continuously and to provide rapid support systems, professional psychological counseling, and crisis interventions (Chen et al., 2020).

The limitations of the study were that all the participating doctors and nurses were interviewed by telephone because there was strict lockdown in Karachi, and there was no physical access to the hospitals. Therefore, the non-verbal expressions was not observed and recorded. The semi-structured guide was not pretested, but the researchers were well trained in conducting telephone interviews before this study. Secondly, the study employed a theoretical sampling where every new interview has given an idea of the new questions that need to add until the researchers reached theoretical saturation point.

## Data Availability Statement

The datasets presented in this article are not readily available because of the confidentiality agreement with respective hospitals and the participants.

## Ethics Statement

The studies involving human participants were reviewed and approved by Ethical Review Board National College of Business Administration & Economics (NCBA&E). Written informed consent for participation was not required for this study in accordance with the national legislation and the institutional requirements.

## Author Contributions

AR and SM formulated the idea of this urgent research, due to emerging and rapidly evolving situation of COVID-19 in Pakistan; made substantial contributions to the conception of the study; and shared and discussed their idea with MZ, who is an MBBS doctor, to check the ground reality and possibility for the conduct of study. MZ assessed the overall situation, verified the facts, and contributed to data collection process by obtaining the permission from relevant hospital administrations through proper channels. AR and DN conducted the telephone interviews, performed the translation and then transcription simultaneously. AR and AH analyzed qualitative data to get emerging themes. AK, NAR, and HA participated as experts and reviewing analysis process and findings. NA provided feedback on each step of the investigation. AR, SM, and DN took lead to draft the *Introduction* and *Materials and Methods*. AK provided tremendous help in drafting the *Abstract* and *Discussion and Concluding Remark*. All the authors were significantly involved in the investigation to contribute to the knowledge in this study.

## Conflict of Interest

The authors declare that the research was conducted in the absence of any commercial or financial relationships that could be construed as a potential conflict of interest.
